# Additional tools to boost internal medicine residents’ evaluations

**DOI:** 10.5116/ijme.5c6c.358f

**Published:** 2019-02-28

**Authors:** Yousef Abdel-Aziz, William R. Barnett, Nezam Altorok, Ragheb Assaly

**Affiliations:** 1 Internal Medicine, University of Toledo, Toledo, OH, USA

**Keywords:** Additional tools, internal medicine, residents’ evaluations

## To the Editor

In 2002, the Accreditation Council for Graduate Medical Education (ACGME) identified six core competencies to be used by graduate medical education programs to evaluate their residents-in-training. The six ACGME core competencies include medical knowledge, patient care and procedural skills, practice-based learning, systems-based practice, professionalism, and interpersonal communication. Each competency is composed of different milestones that residents are required to master at key stages in their medical training. Furthermore, an evaluator chooses the best representation of the resident’s current performance from descriptive narratives, which are rated on a spectrum of critical deficiencies to aspirational. In a cross-sectional study done in 2013-2014, milestone-based ratings correlated with the American Board of Internal Medicine (ABIM) certification examination scores, which may support the validity of a milestone-based assessment tool.^1^ However, it is unclear how these ratings can detect critical deficiencies in trainees, as the milestones only provide a framework to assess residents in key areas of physician competency and not across the entire breadth of the six domains.^2^ The aim of our article was to share our Internal Medicine residency program’s experience in using additional tools beyond descriptive narratives that can boost the evaluation of Internal Medicine residents.

To evaluate a resident’s performance, the ACGME asks the evaluator to assess whether the resident possesses the requisite medical knowledge to provide care and use proper diagnostic testing. It can be difficult to assess a resident depending solely on a clinical rotation, which could be adversely affected by subjective bias. To assess medical knowledge of our residents, our program uses a scoring system for the milestone evaluation by peers, fellows, and faculty members, the in-training exam score, and a six-month average score of the monthly test assessment by our program for all residents. The monthly test questions are used to query the resident’s knowledge about topics that are related to previous month’s didactics. In addition, our program provides residents access to a trackable question bank and residents are expected to complete a targeted program percentage over 6 months. In a cohort study, the monthly test, core competencies, and the in-training exam scores were analyzed for graduates who completed the American Board of Internal Medicine (ABIM) examination from 2010 to 2013. Results showed that a monthly test is a valuable tool to predict performance at the ABIM examination and to identify problem residents, who may need early remediation during their residency. Additionally, the monthly test along with the in-training exam has helped our residency program to highlight areas that requires more focus in the didactic curriculum.^3^

To assess patient care and procedural skills competency based on the ACGME milestones, the resident should be gathering essential and accurate information, developing an appropriate care plan, and managing patients with progressive responsibility and independence. Our program uses a scored evaluation of each resident by peers, fellows, and faculty members during each rotation. Also, there is a target number of required procedures (central line, arterial and venous blood sample withdrawal) that should be achieved during each 6 months. Our institution has an integrated simulation center to improve their procedural skills, interprofessional communication, training outcomes, and patient safety. Our residents are required to attend sessions at the simulation lab every 3 months related to upcoming rotations. This includes a mega code with cardiac arrest simulation, thoracentesis, abdominal paracentesis, central line placement, and bedside ultrasound use. Also, our evaluation of a resident includes a given score depending on the ABIM Mini-Clinical Evaluation Exercise for trainees, which is intended to facilitate formative assessment of core clinical skills.

Assessment of practice-based learning (PBL) is the most challenging among the six competencies due to the lack of published tools. ACGME milestones suggest that residents monitor their own practice with a goal of improvement, learn by performing audits, and be open to feedback. The tools to assess PBL has multiple aspects, such as evaluations by peers, fellows and faculty, participation in at least one quality improvement project, scholarly activities (i.e., publications, abstracts, and conference presentations), chart audit of the resident’s notes, and the autopsy learning module. We use an autopsy learning module as a meaningful tool for practice-based learning by means of self-reflection of clinical experience.4 In addition, we use monthly morbidity and mortality sessions delivered by senior residents to help self-reflection of their clinical experience. Another tool is the evaluation of quality measures associated with the resident’s continuity clinic, which include the assessment and control of diabetes, body mass index, hypertension, and other preventive medicine measures. Also, our senior residents participate in peer review analysis of mortality cases during their ambulatory rotations. This will expand their horizons in data analysis, root cause analysis, and improve their self-learning experience.

The last three core competencies consist of system-based practice (SBP), professionalism, and communication. When evaluating a resident’s SBP competency, ACGME suggests they should be working effectively within an interprofessional team, recognizing system errors, and identifying high-cost care. SBP tools are composed of evaluations by peers, fellows and faculty staff, in addition to participation in different hospital and residency committees. Evaluation of professionalism by ACGME milestones entails respectful interactions with patients, families, and team members, acting responsible, and behaving ethically. The professionalism competency is assessed using evaluations by peers, fellows, and faculty plus didactic lecture attendance. Also, any professionalism letter issued to the resident will be taken into consideration. Lastly, residents are evaluated on milestones, such as communicating with patients and caregivers alongside communicating effectively with team members. The assessment tools for interpersonal and communication skills consists of milestone evaluations by peers, fellows and faculty, nurses, clerks, and students.

Relying on the evaluation of a milestone based on a descriptive narrative has its subjective flaws. In this article, we introduced our own residency program's experience using additional tools for assessment besides ACGME milestones. Other residency programs have suggested that ACGME milestones be used as both as an assessment tool and developmental blueprint, which could create better, more individualized education plans.^5^ Likewise, the process of resident assessment is complex, but should be completed on a semi-annual basis and comprise both quantitative and qualitative data.^6^ We believe that additional tools beyond descriptive narratives can boost the assessment of Internal Medicine residents.

### Conflict of Interest

The author declares that there are no conflicts of interest.
